# Editorial: Building Strategies for Porcine Cancer Models

**DOI:** 10.3389/fgene.2018.00377

**Published:** 2018-10-02

**Authors:** Tiago Collares, Fabiana K. Seixas, Laurie A. Rund, Lawrence B. Schook

**Affiliations:** ^1^Laboratory of Cancer Biotechnology, Biotechnology Postgraduate Program, Technology Development Center, Federal University of Pelotas, Pelotas, Brazil; ^2^Animal Science, University of Illinois at Urbana-Champaign, Champaign, IL, United States; ^3^Department of Radiology, University of Illinois at Chicago, Chicago, IL, United States

**Keywords:** oncopig, pig models, genome editing technologies, swine, cancer models

## Introduction

Pigs are proven models for biomedical studies due to their anatomical and physiological similarities with humans. Pig models have increasingly been validated for mimicking human diseases (Prather, 2013). The sequencing of the swine genome has demonstrated genetic similarities with humans (Groenen et al., 2012).

These considerations led Schook et al. (2015) to develop a transgenic swine model of cancer—the oncopig cancer model (OCM). In addition, recent transcription profile studies of the oncopig's soft tissue sarcoma cells demonstrated altered TP53 signaling, activation of Wnt signaling, and epigenetic reprogramming—all transcriptional features found in human soft tissue sarcoma tumors (Schachtschneider et al., 2017a).

In addition to sarcomas, other cancers have been developed to date using the OCM platform: hepatocellular carcinoma (HCC) and pancreatic cancer (PA) (Schachtschneider et al., 2017c). Oncopig HCC acquired histopathological characteristics similar to human HCC [arginase expression and alpha-fetoprotein (AFP) secretion] and formed tumors after autologous injection (Schachtschneider et al., 2017b). In addition, human HCC transcriptional characteristics were also detected in porcine HCC (Schachtschneider et al., 2017b). In a review recently published by Segatto et al. pigs were proposed as a complementary platform for the discovery of new therapies against cancer through phenotypic screening of compounds, due to the metabolic, physiological, and genetic similarities of pigs with humans (Segatto et al., 2017).

On the research topic “Building strategies for porcine cancer models,” seven papers have been published, with contributions from 40 authors from different institutions around the world, with a focus on molecular and cellular approaches for the development of porcine cancer models.

Watson et al. have reviewed the limitations of rodent models of cancer through a comparison of knockout mouse models to human patients. The authors have highlighted the advantages of using the swine as the biomedical model for cancer research, reviewing special aspects from the swine genome sequence and potential homologies to the human genome. They present the advantages of targeted gene editing using custom endonucleases—specifically TALENs and CRISPRs—and transposon systems, to make novel pig models of cancer with broad preclinical applications.

Schook et al. have discussed genetic modification technologies successfully used to produce porcine biomedical models, in particular the Cre-Lox system as well as the major advances and perspectives of the CRISPR/Cas9 system, highlighting its capacity to induce mutations at a chosen time and space, a characteristic that is especially important when creating genetic models of cancer. Recent advancements in porcine tumor modeling and genome editing will bring porcine models to the forefront of translational cancer research.

Duran-Struuck et al. have reviewed the limitations in using rodents to model human diseases, including the large differences in size, anatomy, physiology, drug metabolism, chromosome structures, and genetics. The authors have highlighted an exciting perspective from their experience with myeloid and lymphoid tumors in major histocompatibility complex characterized miniature swine and future approaches regarding the development of a large animal transplantable tumor model. This work covered the incidence of chronic myeloid leukemias in swine, highlighting the importance of genetic studies of these tumors that can provide a new platform for the development of novel human therapeutics for genetically similar human tumors. Moreover, they discussed the potential of swine models to study post-transplant lymphoproliferative disease (PTLD), since immunosuppressed swine present several characteristics that closely resemble human PTLD. The authors reported their attempt to establish an immortal cell line that could induce PTLD when inoculated into the same inbred line animals.

Gutierrez et al. have presented the potential applications and advantages of using pigs, particularly minipigs, as indispensable large animal models in fundamental and clinical research, including the development of therapeutics for inherited and chronic disorders including cancers. The authors have reviewed examples of naturally occurring conditions in pigs that closely mimic those affecting humans (like malignant spontaneously regressing melanomas, dwarf phenotype, and ventricular septal defect) as well as examples of induced swine models of diseases (for type I diabetes, obesity and metabolic syndromes, and liver cancer models) and established engineered pig models (for cystic fibrosis, heart arrhythmias, xenotransplants, and several types of cancer).

Overgaard et al. have shared original research regarding the use of pigs as a large animal model for cancer vaccine development. Their work demonstrated that the pig model is highly appropriate for addressing the questions related to optimal adjuvant composition and vaccine formulations. The authors investigated whether it is possible for pigs to generate immune responses to cancer antigens RhoC and IDO, using three different adjuvants (CAF09, CASAC, or ISA 51 VG). The results showed that all adjuvants tested were capable of generating some cytotoxic T lymphocytes (CTL) response to the cancer antigens following peptide immunization. These findings support the further use of the pig as a large animal model for vaccine development against human cancer.

Clark et al. have demonstrated that BRCA1 inactivation in pig cells promotes transformation and thus serves as a model for human cancers. The authors established an immortalized porcine breast cell line and stably inactivated BRCA1 using miRNA. The cell line developed the characteristics of breast cancer stem cells and exhibited a transformed phenotype. These results validate the concept of using pigs as a model to study BRCA1 defects in breast cancer and establish the first porcine breast tumor cell line.

Bourneuf has reviewed melanoma genetics, discussing some of the most common mutations found in this type of tumor in humans. Examples of melanoma animal models have also been discussed, with emphasis on the porcine MeLiM model. This work suggests that the spontaneous tumor progression and regression occurring in these models could shed light on the interplay between endogenous retroviruses, melanomagenesis, and adaptive immune response.

## Conclusion

Combined, the studies on this research topic have demonstrated that pigs are proven useful models for cancer studies including in (1) the development of genetically engineered pigs by using different technologies like TALENs, CRISPRs, transposons, and the Cre-Lox system; and (2) models for myeloid, lymphoid, breast, and melanoma cancers. Thus, the porcine genome sequence coupled with somatic cell cloning has led to the development of innovative porcine cancer models to support translational cancer research.

## Author contributions

TC and FS are responsible for organizing the materials and writing this editorial. LR and LS are responsible for proofreading this editorial.

### Conflict of interest statement

The authors declare that the research was conducted in the absence of any commercial or financial relationships that could be construed as a potential conflict of interest.
